# Booster or Killer? Research on Undertaking Transferred Industries and Residents’ Well-Being Improvements

**DOI:** 10.3390/ijerph192215422

**Published:** 2022-11-21

**Authors:** Xuhui Ding, Yong Chen, Min Li, Narisu Liu

**Affiliations:** 1 School of Finance and Economics, Institute of Industrial Economics, Jiangsu University, Zhenjiang 212013, China; 2 School of Management, Hebei University, Baoding 071002, China; 3 School of Economics and Management, Jiaying University, Meizhou 514015, China

**Keywords:** inter-regional industrial transfer, residents’ well-being, industry undertaking place, booster or killer, spatial econometric model

## Abstract

Inter-regional industrial transfers would change the economic, societal, and ecological environment of the undertaking area profoundly. Some experts have recognized the ecological and environmental problems caused by industrial transfers. However, there are few studies on whether undertaking an industrial transfer will ultimately improve the well-being of residents. There is a strong application value for exploring this issue under the domestic cycle in China. This paper uses the shift-share analysis method to measure China’s inter-provincial industrial transfer from 2004 to 2019. According to the subjective and objective indicators, the article measures the level of residents’ well-being. A spatial econometric model is used to empirically test the impact of undertaking transferred industries on residents’ well-being and its mechanism. The results show that: 1. There is a significant spatial positive correlation between the well-being of residents at the national level. The empirical results also indicated significant spatial correlations at the level of the three major economic belts in the east, central, west, and northeast; 2. From the perspective of China as a whole, the inter-regional industrial transfer improved the well-being of the residents significantly, but the indirect negative effect reduced the total effect; 3. From the regional perspective, undertaking a transferred industry could significantly improve the well-being of residents in the central and eastern regions. However, in the northeast and western regions, it showed a serious negative effect. We should enhance the orderly transfer of industries deeply, considering the ecological and environmental capacities of the undertaking area fully and strictly limiting the inter-regional transfer of polluting industries. Only in this way could the government improve the well-being of residents in the industrial transfer-out areas and undertake areas effectively.

## 1. Introduction

**Figure 1:**
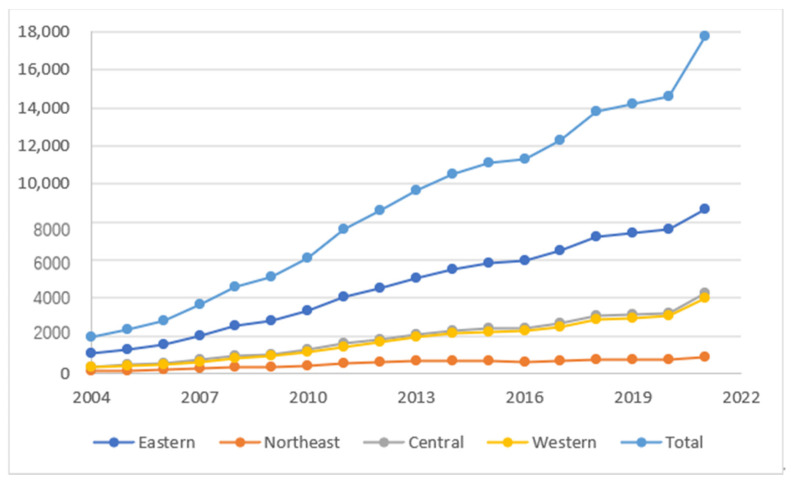
Trends of the GDP in the four regions from 2004 to 2021. Source: China Statistical Yearbook.

A better life has always been the aspiration of people. With continuous economic growth and rapid industrial development, improving the well-being of residents has become the starting point for relevant policy formulations. Over the past 40 years of reform and opening, Eastern China has actively undertaken foreign industrial transfers to achieve an economic take-off. In 2021, the per capita GDP of the Jiangsu province reached USD 18,920, while the per capita GDP of the Gansu province was only USD 5636 in the same period. The huge difference in income between the eastern and western provinces should be given attention. This is a real problem facing China’s common prosperity and overall happiness ideals, and it also needs the guidance of theoretical research urgently. Figure 1 is China’s 2004–2021 sub-regional economic growth line chart. It can be seen that there are significant differences between the east, middle, west, and northeast, both in volume and growth rate. Inter-regional industrial transfers to the central and western provinces and cities have become an important path to achieve common prosperity. It brings capital investment, productive technology, and enterprises to the undertaking place, promotes economic growth, and creates a large number of jobs. However, the inter-regional industrial transfer has an impact on the original ecological pattern of the undertaking area, and the diffusion of environmental pollution is easy to cause the pollution refuge effect. The double-edged sword characteristics of industrial transfers are not conducive to the improvement of residents’ well-being [1]. In 2020, the total nitrogen and phosphorus emissions from the wastewater in the central and western regions accounted for 62.66% and 65.34% of the total national emissions, respectively, while the sulfur dioxide emissions in the central and western regions accounted for 72.8% of the total national emissions. Domestic circulation is bound to trigger a new wave of industrial transfers. Researching methods that ensure the improvement of residents’ well-being while undertaking industrial transfers and exploring the internal relationship between the two has strong practical significance and application value. 

## 2. Literature Review and Theoretical Mechanism

### 2.1. Literature Review

Academic research on the issue of industrial transfers was started early. Many classic industrial transfer theories were formed, such as the goose theory, the marginal industrial expansion theory, the international production compromise theory, etc. These theories explain the reason for some countries’ rise. Some scholars interpret the issue of industrial transfers in combination with the trend of globalization. Kurata H. et al. explained industrial transfers from the perspective of the vertical specialization of the north-south trade [2]. They proposed that the decline in trade costs at different production stages would encourage vertical specialization, and the final product would be transferred to southern countries. Soberg P.V. found that the essence of industrial transfers is often technology transfer [3]. The cooperation between industries and capital could promote the high-quality development of regional economies. Gopalan S. et al. explored the issue of global value chain participation from the perspective of enterprise digitization and proposed that the rapid development of electronic communication technology prompted multinational companies to outsource their production activities to emerging industrial countries [4]. After China gradually became the world’s manufacturing factory, strategies, such as the development of the western region and the rise of the central region, were put forward one after another. Scholars began to focus on inter-regional industrial transfers and conducted many useful explorations in terms of scale measurement, spatial characteristics, and realization paths [5,6,7]. Tan G. et al. conducted a two-dimensional analysis of the driving mechanism and preferred areas of industrial transfers [8]. Their team refined the evolution path of China’s industrial transfer and the interaction between the symbiotic system and the symbiotic environment of the industrial transfer. Ang Y.Y. believed that China’s industrial transfer had its own characteristics, such as its more obvious domestic industry goose array form [9]. The western region often has to undertake too many pollution enterprises. Zhang Y.G. combined the gradient transfer theory with the multi-regional input-output model. He put forward the method of judging the industrial transfer mode and summarized the five industrial transfer modes, including agglomeration, diffusion, undertaking, backflow, and substitution [10].

With the rise of international and inter-regional industrial transfers, experts began to pay attention to the ecological and environmental problems caused by industrial transfers. The pollution haven hypothesis and other related theories came into being [11,12,13]. Candau F. and Dienesch E. proposed that the pollution paradise hypothesis does exist in some cities in Europe. The expansion of foreign direct investments and trade openness has led to increased pressures on local environmental degradation [14]. Zhang Z.H. et al. found that air pollution from industries tends to reduce residents’ life expectancy and has significant spatial spillover effects. They confirmed a significant positive correlation and spatial spillover effect between social status and life expectancy [15]. Early research on well-being focused on areas such as economics and sociology. As ecological degradation and environmental pollution became prominent, scholars paid attention to the contributions of the ecological environment to the well-being of residents [16,17]. Dai Y. et al. constructed an evaluation system of urban ecological governance based on the perspective of residents’ well-being. They proposed that environmental regulations could improve ecological efficiencies by regulating the transferred industries [18]. Inter-regional industrial transfers have multiple effects on the economy, society, and environment of the undertaking area, thus boosting or reducing the improvement of residents’ well-being in the undertaking area. However, little literature has focused on the specific impact and path of inter-regional industrial transfers on residents’ well-being [19]. Under the domestic circulation with the inter-regional industrial transfer as the core, this article adopts a scientific measurement of the inter-regional industrial transfer and residents’ well-being index. Meanwhile, it uses spatial econometric and moderating effect models to explore the change and influence path of the residents’ well-being effect caused by the inter-regional industrial transfer. The article puts forward some practical and feasible policy suggestions, which have strong practical significance and application value for boosting China’s domestic circulation and enhancing residents’ happiness.

### 2.2. Theoretical Mechanism

The core of well-being is good living conditions, including utility satisfaction, life quality, people’s satisfaction, etc. Over the past 40 years of reform and opening in China, the concept of development has been broadened from economic growth to various fields of economy and society. People’s eagerness for a better life has become more extensive and specific. Essentially, an industrial transfer is the result of the transformation of regional industrial competitive advantages. It is the process of optimizing the allocation of resources and capital in a wider range, and it is also a common method of division and cooperation among industries. According to the theory of the inter-regional economic development relationship, the labor-intensive and energy-oriented industries in the eastern regions of China have gradually lost their competitive advantages. Due to rising factor costs and other reasons, the thrust of regional industrial structure adjustments exists. Coupled with the local government’s preferential attracting policies, the central and western regions are the advantageous production areas of the eastern backward production capacity. Chinese central and western regions are forming dominant production areas to attract inter-regional industrial transfers. Through the introduction of equipment and talent, each region realizes the win-win development of enterprises and regions and ultimately optimizes social welfare. In general, industrial transfer in China is a market behavior guided by policy and spontaneous enterprises. The ways to affect the well-being of residents mainly include the following two aspects.

On the one hand, the direct impact of industrial transfers on the well-being of residents exists. The direct impact is mainly a positive promotional effect, including a negative offset effect. First, the undertaking of transfer industries could effectively improve the income of local residents [20]. The increase in income could improve people’s living standards and meet diverse material and cultural needs. The satisfaction of utilities could greatly enhance the perception of happiness among residents [21]. Secondly, the undertaking of the transfer industry could expand the employment demand of the region. Employment is people’s biggest livelihood. Focusing on stable employment as the goal orientation is also the proper meaning of “six stability” in China. By increasing fiscal expenditure and implementing encouraging science and technology policies, local governments attract competitive enterprises to build and operate in order to improve the job demand of enterprises as well as high regional employment [22]. Thirdly, the undertaking of the transfer industry reduces the environmental level of the transferred area. The vast majority of industrial transfers are dominated by manufacturing at present, which has an inevitable impact on the local environment [23]. With the introduction of the concept of green development, physical and mental health was gradually placed in a more prominent position, which is also a part of the research that cannot be ignored.

On the other hand, industrial transfers also have indirect impacts on the well-being level of residents in the undertaking area. These effects mainly manifest in the adjustment of the industrial structure, the improvement of the urbanization rate, and the transfer payment of the government [24,25]. The undertaking of transfer industries has improved the regional industrial structure, which not only increases the vitality of the market but also promotes the high-quality development of the economy. At the same time, the industrial structure and people’s well-being show a strong coupling effect under the influence of financial constraints [26]. Gresik T.A. and Osmundsen P. believed that while undertaking industrial transfers could increase employment, it could also increase the wage income of manufacturing personnel and the tax revenue of the government of the undertaking area [27]. Davidson R. et al. showed that the government’s measures to regulate the gap between the rich and the poor, as well as maintain social fairness and justice, could effectively enhance the happiness of residents. They also point out that the size of the government plays a decisive role in it [28]. When the local financial situation is better, there is still a surplus in addition to the allocation of basic economic constructions, which gives local governments more autonomy to use financial funds for the construction of people’s livelihoods. The government could introduce policies to actively benefit the people, effectively improve the quality of life of low-income groups, and promote the improvement of residents’ well-being.

Judging from the above discussion, the complexity of undertaking an industrial transfer effect under the influence of market mechanisms becomes more confusing; the new development pattern of the government also easily falls into a dilemma game. Would an industrial transfer effectively improve the well-being of local residents? What should the government do when “GDP-only” no longer holds much weight in the promotion tournament for officials? How do we reduce its negative effects while undertaking industries? This paper introduces environmental regulations as a moderating variable to study its effect on industrial transfers and residents’ well-being. The implementation of environmental regulations not only regulates the operation of the transfer industry but also increases the happiness of residents [29,30]. The government’s increased investment in environmental regulation could correct the pollution effect of the transfer industry effectively. The industrial structure of the receiving area could be upgraded and rationalized by strict regulations [31]. This paper argues that under the background of the great cycle policy, enterprises adjust their location to promote the optimization of local industrial structures constantly. In this process, industrial transfers play a role in improving the well-being of residents through direct effects, spatial spillover mechanisms, and regulatory mechanisms of environmental regulations.

## 3. Materials and Methods

### 3.1. Research Methods and Models

The local-neighboring effect of industrial transfers has always been a hot research topic in academia. Liu Y.J. et al. used the shift-share method and spatial panel model to analyze the impact of industrial transfers on the surrounding haze empirically [32]. At the same time, some invisible industrial transfers, such as the flow of industrial personnel, would also significantly affect the economic structure of neighboring areas [33]. In order to fully consider the impact of undertaking an industrial transfer on residents’ well-being, this paper draws on the research of Xu J.et al. and uses the spatial model considering the time-space effect to evaluate the industrial transfer [34]. Spatial econometric models mainly include the spatial lag model (SLM), the spatial error model (SEM), and the spatial Durbin model (SDM). Since the optimal model has not been determined yet, the general form of the spatial model could be established as Formulas (1) and (2). The level of residents’ well-being is marked as $y_{it}$; $x_{it}$ for each influencing factor; τ, ρ, δ and λ are coefficient terms; $w_{ij}$ is the spatial weight matrix. This study uses two spatial weight matrices, economic distance ($w_{1}$), and geographical distance ($w_{2}$), to study the spatial characteristics of residents’ well-being. $\mu_{i}$ and $\nu_{i}$ are the time point effect and individual effect, respectively; $\varepsilon_{it}$ is a random item. When τ=ρ=δ=λ=0 and λ≠0, we select SEM; when δ=λ=0 and τ≠0 or ρ≠0, SLM is selected; when λ=0 and τ≠0 or ρ≠0 or δ≠0, the SDM is selected. The selection of the specific model still needed to be determined with the test results.

(1) $$y_{it}=ty_{i,t-1}+r\sum_{j=1}^{N}w_{ij}y_{jt}+bx_{it}+d\sum_{j=1}^{N}w_{ij}x_{ij}+m_{i}+n_{i}+e_{it}$$

(2) $$\varepsilon_{it}=\lambda \sum_{j=1}^{N}w_{ij}\varepsilon_{jt}+\varphi_{it},\underset{}{}\varphi_{it}∼N(0,\sigma_{it}I_{n})$$

### 3.2. Variable Setting

The residents’ well-being level was denoted as “**wal**”. On the one hand, the improvement of residents’ well-being comes from the improvement of objective conditions, which are often related to the quality of life [35]. On the other hand, the measurement of well-being should also integrate the subjective well-being of residents [36,37]. The article referred to the HDI index released by the United Nations in 1990 to measure the level of objective well-being [38], which consists of three aspects: health and longevity, decent living, and access to knowledge. Considering the availability of data in each province, this paper used the improved HDI index system according to the research by Xiao Q.L. et al. [39]. The number of doctors per 10,000 people was used instead of the per capita life expectancy when measuring health and longevity. The average number of years of education was derived from the total number of years of education aged 6 years and over divided by the total number of inhabitants aged 6 years and over. The total number of years of education was calculated as a weighted average of the number of years received by the people in primary, lower secondary, upper secondary, and higher education. In addition to the health index, both the income index and education index were obtained by normalizing the relevant indicators. The objective development index of the residents was calculated with the geometric mean method using the health, income, and education indexes. The Formula is (3).

(3) $$RDI={\left(health\times education\times income\right)}^{\frac{1}{3}}$$

With reference to He Y.H.’s method of measuring subjective well-being, this paper studies the subjective well-being of residents by means of a social survey [40]. The strength of well-being was divided and assigned to different levels, and the scores of the interviewees in different regions were statistically classified. The weighted average of the scores of all respondents was the subjective well-being index, which was calculated with Formula (4), where SWBI is the subjective well-being index of the residents in the undertaking area; n is the grade of happiness; m is the total number of respondents; and $p_{i}$ is the number of respondents with an i level of happiness; the higher the i value, the stronger the subjective well-being of the respondents. The research on the well-being of the residents in the undertaking area needed to combine the subjective and objective aspects to be more comprehensive. The geometric average method takes into account the incomplete substitution of the subjective and objective well-being levels and measures the well-being level of the residents from these two dimensions. The geometric average of the two types of indexes better reflects the well-being level of the regional residents. The calculation Formulas are (4) and (5):

(4) $$SWBI=\sum_{i=1}^{n}\frac{i}{n}\times \frac{p_{i}}{m}$$

(5) $$WBI={\left(RDI\times SWBI\right)}^{\frac{1}{2}}$$

The industrial transfer variable was “**indus**”. In order to distinguish the impact of undertaking an industrial transfer on the well-being of local residents in different regions, this paper divided 30 provinces and cities into eastern, northeastern, central, and western regions. Referring to the research by Cheng A.H. et al., the deviation share method was used to build Formulas (6)–(8), where $X_{ij}$ is the initial value of the industry i in the region j(i=1,2,⋅⋅⋅,S;j=1,2,⋅⋅⋅,R) [41]. $X_{ij}^{’}$ is the value of this variable at the end of the defined term. The change in the economic variable over this time period was measured using Formulas (9) and (10). $X_{ij}r$ is the share component, which refers to the added value of the industry in the undertaking area according to the average growth rate of the national industry. $X_{ij}\left(r_{i}-r\right)$ is the structural deviation component, which refers to the influence of the deviation of the growth value caused by the difference between the industrial growth rate and the national average growth rate of the industry in the undertaking area. $X_{ij}\left(r_{ij}-r_{i}\right)$ is the competitiveness deviation component, which measures the impact of the actual growth value of the industry in the undertaking area on the industry.

(6) $$\begin{array}{l} QIT=\sum_{j=1}^{R}Xij\left(rij-ri\right)\\ \;\;=\left[Xi1\left(ri1-ri\right)\right]+\left[Xi2\left(ri2-ri\right)\right]+\cdots +\left[XiR\left(riR-ri\right)\right] \end{array}$$

(7) $$r_{i}=\sum_{j=1}^{R}\left(X_{ij}^{\prime }-X_{ij}\right)/\sum_{j=1}^{R}X_{ij}$$

(8) $$r_{ij}=\left(X_{ij}^{\prime }-X_{ij}\right)/X_{ij}$$

(9) $$r=\sum_{i=1}^{S}\sum_{j=1}^{R}\left(X_{ij}^{\prime }-X_{ij}\right)/\sum_{i=1}^{S}\sum_{j=1}^{R}X_{ij}$$

(10) $$\Delta Xij=X_{ij}^{\prime }-X_{ij}=X_{ij}r+X_{ij}(r_{i}-r)+X_{ij}(r_{ij}-r_{i})$$

The environmental regulation variable was denoted as “**regul**”. Environmental regulations counterbalance the adverse effects of undertaking an industrial transfer on regional living environments and promote the standardized operation of a market economy. Lee et al. found that there is a significant positive correlation between residents’ subjective well-being and the level of environmental regulation [42,43]. On the one hand, environmental regulations improve the level of enterprise innovation. On the other hand, it improves the health level of residents, and the health effect of the environmental regulation is stronger than the innovation effect. Based on the research by Ren X.S. et al., this paper collected data on the waste emissions in each province and used the entropy method to calculate the comprehensive index of environmental regulations to measure the level of environmental regulations [44,45].

Control variables. Referring to the idea by Chen et al. [46,47,48,49,50,51,52,53], the following control variables were selected: 1. Unemployment (**unemp**), the unemployment rate was used to characterize the employment effect of happiness.; 2. Per capita consumption (**consum**), we used the proportion of the per capita consumption to per capita GDP to measure the consumption expenditure of each region.; 3. R&D intensity (**innova**), this paper chose the ratio of regional R&D investments to the GDP as the proxy variable of R&D intensity.; 4. Government intervention level (**gov**), we used the ratio of the total fiscal transfer payment and social security input to the GDP as the proxy variable of the government intervention level.; 5. Openness (**open**) was measured with the proportion of FDI in GDP.; 6. The Theil index (**tele**) used the concept of the entropy method to measure regional income inequality.; 7. Economic efficiency (**lumin**) was measured with the night light index.; 8. Industrial structure (**stru**) was measured with the proportion of the total output value of the secondary industry in GDP. In addition, the article carried out Z-score standardization for the non-ratio variables.

### 3.3. Data Description and Descriptive Statistics

Considering the continuity and availability of the industrial transfer data, the research samples were panel data from 30 provinces and cities in China from 2004 to 2019. The reason for using 2004 as the starting year was that the social survey on subjective well-being was launched that year, and the unpublished data in 2019 were predicted by modeling. In view of the integrity of the data, this study did not include Hong Kong, Macao, Taiwan, or Tibet. The main data were derived from statistical yearbooks and statistical bulletins from each province in China. The average years of education data for calculating the education index in the HDI sub-index were derived from the “China population and employment statistical yearbook”. The industrial transfer data were derived from the “China industrial statistical yearbook”, and the night-time light index was derived from the Wind database. The data of the subjective well-being survey were derived from the China general social survey (CGSS). To deal with some of the missing values, the article used an interpolation method. This article uses Stata 16.0 developed by the U.S. Computer Resource Center to process and analyze data. Descriptive statistics of each variable are shown in Table 1.

## 4. Empirical Results and Analysis

### 4.1. Overall Analysis

**Figure 2:**
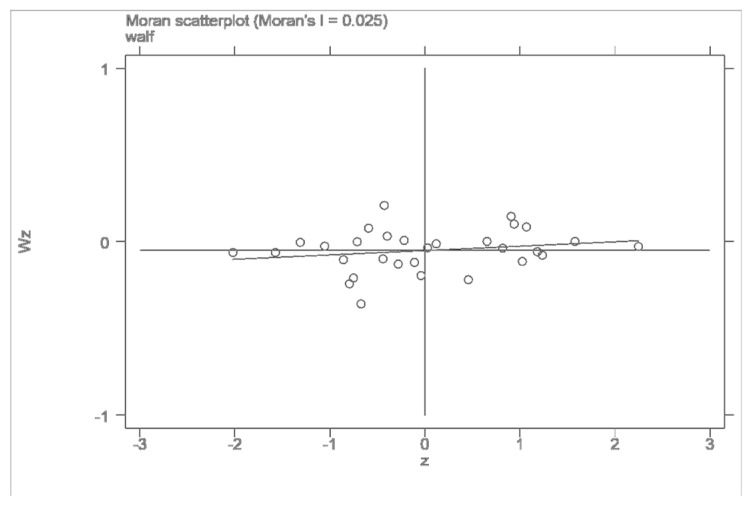
Local Moran scatter plot of residents’ well-being in 2005.

**Figure 3:**
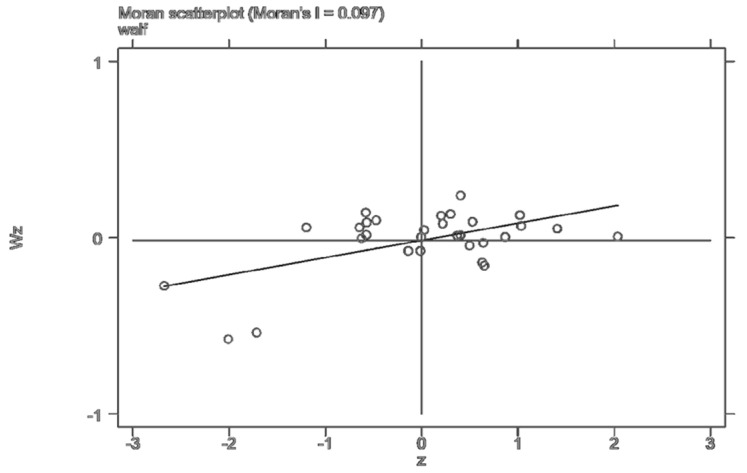
Local Moran scatter plot of residents’ well-being in 2012.

**Figure 4:**
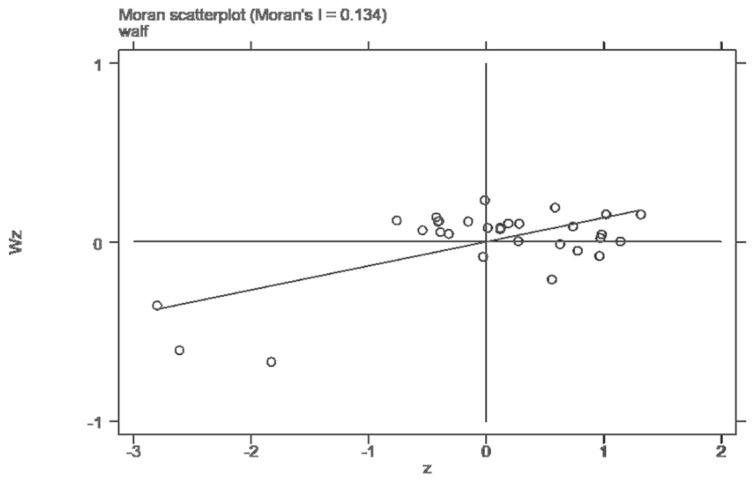
Local Moran scatter plot of residents’ well-being in 2018.

Since the industrial transfer as an explanatory variable had a significant time-space effect, it could not meet the premise hypothesis of the traditional econometric model. This paper used the OLS-[SAR\SEM]-SAC-SDM method for estimation. Firstly, Moran’s I was used to test the spatial dependence of the data, and the results are shown in Table 2. It can be seen that Moran’s I of the Chinese provincial residents’ well-being level from 2005 to 2008 was significantly positive at the level of 10%. Moran’s I of the well-being level of the provincial residents from 2009 to 2019 was significantly positive at the level of 1%, indicating a significant spatial positive correlation and a certain cluster trend in the well-being of people’s livelihood across the country. At the same time, the local Moran scatter plots of 2005, 2012, and 2018 were drawn based on the weight matrix (Figure 2, Figure 3 and Figure 4). It can be found that Moran’s value of the three characteristic years was greater than 0.1. Most of the provinces were in the first and third quadrants, indicating that the spatial agglomeration model of the residents’ well-being had a strong proximity effect. These figures also confirmed the global Moran index’s judgment that there was a positive spatial effect on the level of residents’ well-being. The differentiation pattern of residents’ well-being in each year remained stable and had a strong spatial correlation.

This study used a spatial econometric model to analyze this effect quantitatively. Table 3 shows the regression results of the different models. The results show that the $R^{2}$ value of the SDM model is higher than that of the other two types of spatial models, and the fitting effect is the best. Compared with the other models, the estimated results of each variable in the SDM model changed, and the fixed effect model was selected, in which the impact of economic efficiency and economic structure on the well-being of residents was no longer significant. It is obvious that large spatial factors affected the estimation results of each variable. All three spatial models suggested that undertaking an industrial transfer has a negative correlation with residents’ well-being at the national level, which is supported by the pollution haven effect [54,55]. In underdeveloped areas, undertaking an industrial transfer is an important part of improving local residents’ income, which plays an irreplaceable role in regional economic growth and improving residents’ employment. However, the damage to the ecological environment caused by the influx of excessively polluting enterprises often causes residents to be miserable, especially in the western region with weak environmental regulations [56]. The spatial spillover coefficient of undertaking an industrial transfer is positive. On the one hand, according to the flying geese theory, the industry itself has a strong external effect, and the transfer of regional industries upgrades the local industries in turn [57]. On the other hand, under the rising environmental regulation standards of local governments, enterprises would also carry out “rent-seeking” behaviors passively. The spatial correlation coefficient (ρ) was positive at the level of 1%, indicating that there is a spatial spillover effect on residents’ well-being.

In terms of the control variables, the spatial model corrected the estimation results of the ordinary least squares much better. The impact of the unemployment rate and household consumption on well-being was negative, which could be related to the short-term pressure of the economy. High fixed expenditure often reduces the well-being of residents; Park K.H. believed that consumer heterogeneity is also a key factor affecting well-being [58]. At the same time, income inequality and the degree of opening to the outside world also reduced the well-being of residents in the receiving areas. It shows that the introduction of low-quality foreign capital is no longer suitable for the current development model of China. Residents pay more and more attention to the environment, and fairness and justice are placed in a more prominent position. Technological progress and economic efficiency have significantly improved the well-being of the host region. Mochon F. believed that the use of technology could effectively narrow the gap between urban and rural areas and improve rural revitalization, as shown by an increase in the overall level of well-being [59]. Besides the unemployment rate and government intervention, the remaining variables have significant spatial spillover effects. This shows that in addition to strengthening their own capacity, the provinces should also actively explore the path of coordinated development with the surrounding regions so as to help improve the well-being of the overall residents of the area.

### 4.2. Analysis by Region

**Figure 5:**
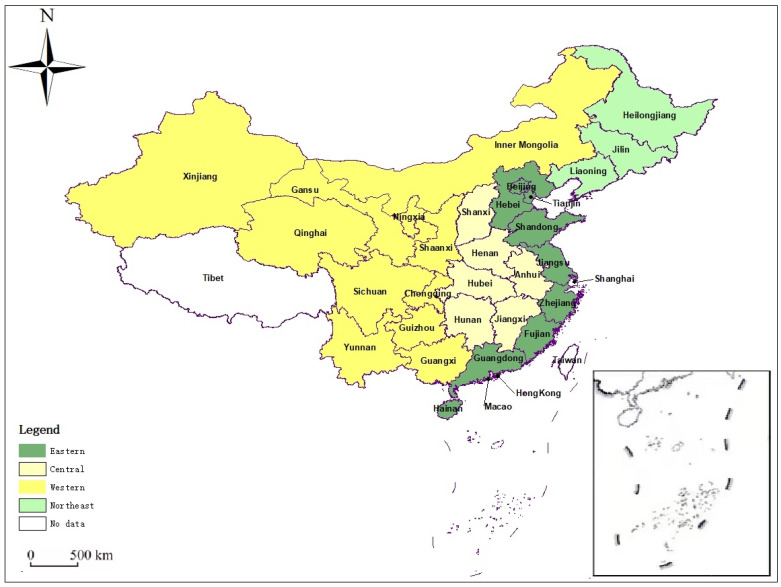
Four main regions of industrial transfer in China.

Different regional situations displayed strong spillover effects of inter-regional industrial transfers. After referring to the relevant research by Wang Y.A. et al., this paper argues that it is necessary to divide the 30 provinces into four regions: eastern, central, western, and northeastern, on the basis of an overall analysis [60]. To deeply explore the relationship between undertaking an industrial transfer and residents’ well-being, the SDM estimation results by region are shown in Table 4. Figure 5 is the map of China drawn by GIS according to the regional division standard of Zhao et al. [61].

For the eastern provinces, the amount of industrial transfer is negative, so the transfer of industries between the regions improved the well-being of local residents. As the main output area of the current industrial transfer, the eastern provinces developed high-tech industries with the transfer of industrial manufacturing. They took the lead in industrial transformations and upgrades. Although there would be some structural unemployment and slowing of economic growth, in order to meet the long-term needs of people’s livelihoods and obtain higher profits in the industrial chain, some backward industries with high pollution and high energy consumption should be abandoned [62]. According to the estimation results of the control variables, the unemployment rate and per capita income inequality as negative indicators affected the well-being level of local residents significantly. At the same time, the government’s adjustment effect on residents’ well-being did not have a significant effect in the eastern region. It can be seen that technological progress has a strong role in promoting the well-being of residents in the eastern region, which is also consistent with the research results of Kenderdine T. and Lan P.Y. [63]. With the gradual advancement of industrial transformation and upgrading in the eastern region, the high-tech industry has gradually become the main force driving the improvement of residents’ well-being.

The three northeastern provinces were also the main transfer-out areas of the industrial transfer. However, their impact on the well-being of residents was opposite to that of the eastern region. Action is needed for the northeast region because of the excessive market competition caused by the serious homogenization of its industry, the solidification of the industrial structure, and the overcapacity of traditional industries caused by the imbalance of the development of the secondary and tertiary industries [64]. The high-quality development of enterprises could be promoted through an industrial transfer and the optimization of market structures. Nevertheless, in the short term, the existing development model that cannot be abandoned is still a persistent disease that hinders the improvement of people’s well-being in the northeast region. In terms of the control variables, the satisfaction of utilities brought by consumer spending has a promoting effect on the level of well-being. During the study period, the degree of income inequality in the three northeastern provinces gradually narrowed; therefore, fair income levels would also significantly improve the well-being of residents. The coefficients of government intervention, economic efficiency, and industrial structure were negative. For the government, this may be related to the implementation of the “last mile”. For the market, as the proportion of the secondary industry in the industrial structure decreases, the industrial structure becomes increasingly reasonable, which is reflected in the increase in the level of residents’ well-being.

As the main undertaking area of the industrial transfer, undertaking an industrial transfer had a strong positive effect on the welfare level of the central region, which is different from the research results of Xu G.Y. et al. [65]. The main reason is that the latter was only analyzed from the environmental effects of the industrial undertaking. In recent years, the developmental potential of the central region has been continuously released. With the accelerated entry of enterprises, the industrial agglomeration effect was enhanced continuously, which promoted the upgrading of local backward industries. With the large-scale influx of enterprises, the continuous reduction of unemployment rates has also improved the well-being level of the undertaking area. It is worth noting that although the central region increases its investment in innovation every year, innovation has a significant inhibitory effect on the well-being of residents in the central region, which is related both to the quality of patents and the time lag of innovation [66,67]. Utility and highly cited patents in the region only account for a small proportion of the total number of patents. It results in the lack of technology to promote industrial upgrading, despite the high investment in technology. The government’s adjustment of income is conducive to the improvement of residents’ well-being, but the effect is not significant.

The western region was also the main area to undertake an industrial transfer, but it was very different from the central region. Undertaking an industrial transfer inhibited the improvement of regional residents’ well-being, but it was not significant, which may be related to undertaking too many high-pollution industries [68]. During the survey period, the unemployment rate, the scale of foreign capital inflow, and the degree of income inequality in the western region continued to decline. The positive coefficient of the unemployment rate shows that employment is not a necessary condition for the improvement of the well-being of the people in the west, which may be related to local customs and an employment mismatch. Less foreign capital inflow increases the level of well-being, which may be related to the fact that the introduction of low-quality foreign capital in the early stage destroyed the local living environment. Fu S.K. et al. believed that when the western region lacks effective environmental regulations as a “pollution haven”, the social cost caused by foreign investments destroying the environment may be far greater than its positive effect on increasing residents’ income [69]. It can be seen that the fiscal expenditure and transfer payments in the central and western regions have not played a good role in improving people’s well-being. This may be related to the implementation of regional government policies and is also affected by the level of regional economic development. The following would further decompose the differential impact of each variable on residents’ well-being, and the results are shown in Table 5.

As far as the central region is concerned, the main transfer-in-place of industries, the direct and spatial spillover effects of undertaking an industrial transfer on residents’ well-being are positive and significant and boosts well-being. The reduction in the unemployment rate improved the well-being of local residents from both local and neighboring areas. Consumption had a significant reverse effect on the level of regional well-being, which was different from the positive effect in the northeast region, which may be related to the higher living pressure in the central region. The direct effects of technological progress reduced the well-being of the residents in the central provinces significantly. The spatial spillover effect on the improvement of well-being was positive but not significant. In terms of the overall effect, technological progress did not affect the well-being of the residents in the region significantly. The spatial spillover effect of government interventions in the central and western regions promoted the improvement of well-being. We should continue to strengthen government functions and improve the level of inter-governmental cooperation. It is worth mentioning that the strong indirect effect of the western region, which was also the place of transfer-in, led to a negative correlation between well-being and industrial undertaking. It indicates that undertaking an industrial transfer is not a universal formula for improving residents’ well-being, and policy-makers should take regional heterogeneity into account [70]. The introduction of high-emission and high-pollution enterprises is the “killer” of local residents’ well-being. The quality of the transfer industry is a part of the local government that cannot be ignored. As a result, inter-regional industrial transfers should be undertaken accurately and guided.

Combined with the results of the regional analysis, it can be seen that due to the existence of regional heterogeneity, the scale of undertaking an industrial transfer has different effects on the well-being of residents in various regions. Among them, the eastern-developed provinces were the main areas of China’s current industrial transfer. As the leader of national economic development, they take the lead in transferring from industrial manufacturing to “change the cages for the birds” for the development of high-tech industries. Their orderly upgrading of industries improved the well-being of local residents. The direct and spatial spillover effects of the northeast region, which is also the place of the industrial transfer, show that the industrial transfer is not ideal for the improvement of residents’ well-being. Since the implementation of the “Northeast revitalization” plan in China, the local industrial structure underwent great changes, but the outflow of talent led to a local inability to support the smooth landing of high-tech industries [71]. The government should improve relevant supporting facilities to avoid labor shortages. As the main industry undertaking region, the late-mover advantage of the central region is constantly emerging. The large-scale transfer of industrial manufacturing in developed regions has brought opportunities for economic development to these regions and has also improved the well-being of the residents. The western region may have undertaken too much pollution industry transfer, resulting in a significant negative correlation between its residents’ well-being and the industrial transfer. At the same time, the impact of other significant variables should also be taken seriously.

### 4.3. Regulatory Effect Test

In order to further study the impact of environmental regulations, undertaking industrial transfers, and residents’ well-being, this paper tested the moderating mechanism, referring to the models constructed by Zhuang et al. [72]. These models also explain the relationship between the improvement of residents’ well-being and the undertaking of an industrial transfer. To examine the relationship among the adjustment variable of environmental regulation, the explanatory variable of undertaking an industrial transfer, and the explained variable of residents’ well-being, the following three regression equations were established. Among them, Y represents the level of residents’ well-being, and the specific calculation results are shown in the variable interpretation part. $\alpha_{1}$, $\alpha_{2}$ and $\alpha_{3}$ are constant terms; Z represents all the control variables; $\varepsilon_{it}$, $v_{it}$ and $\xi_{it}$ are the error terms of the three equations. Specifically, the first equation describes the impact of the independent variables on the dependent variable, ζ reflects the total effect of undertaking an industrial transfer on the well-being of residents. The second equation discusses the relationship between the independent and moderating variables, γ estimates the strength of the connection between undertaking an industrial transfer and environmental regulations. The third equation introduces the adjustment variable on the basis of the first equation. $\theta_{1}$ represents the impact of undertaking an industrial transfer on the well-being of residents after controlling the impact of the adjustment variables; $\theta_{2}$ indicates the impact of environmental regulation on residents’ well-being after controlling the influence of the independent variables.

(11) $$Y_{i,t}=\alpha_{1}+\zeta indus_{i,t}+\psi Z_{i,t-1}+\eta_{t}+\varepsilon_{i,t}$$

(12) $$regul_{i,t}=\alpha_{2}+\gamma indus_{i,t}+\psi Z_{i,t}+\eta_{t}+\upsilon_{i,t}$$

(13) $$Y_{i,t}=\alpha_{3}+\theta_{1}indus_{i,t}+\theta_{2}regul_{i,t}+\eta_{t}+\xi_{i,t}$$

Table 6 is the test of the moderating effect of environmental regulation. The first column of the table shows that the coefficient of the industrial transfer is negative, indicating that undertaking an industrial transfer reduces the well-being of residents. The second column verifies the negative correlation between environmental regulation and undertaking an industrial transfer. After introducing the variables of undertaking an industrial transfer and environmental regulation into the economic model, as shown in the third column of the table, both core explanatory variables are significant at the level of 10%. These results indicate that undertaking an industrial transfer and environmental regulation have a strong impact on the well-being of residents. The significant positive effect of environmental regulation counterbalances the social cost of the industrial transfer and improves the well-being of residents, which is also consistent with the research conclusions of Hamhami A. et al. [73]. The spatial spillover effect of environmental regulation is significantly positive, indicating that environmental regulation in neighboring areas could effectively improve the level of local well-being. The reason could be related to the “competitive” effect of environmental regulation among the local governments [74]. Compared with column 1, the significance and absolute value of the index coefficient of undertaking an industrial transfer in column 3 decreased. In summary, environmental regulation has a fair regulatory effect on all variables. The government should apply environmental regulations to outweigh the shortcomings of the market economy actively and should also be alert to the occurrence of bottom-up competition caused by heterogeneous environmental regulation [75].

### 4.4. Robustness Test

In the robustness test, this paper replaced the spatial weight matrix in the original model with an adjacency matrix. Based on the results of the Hausman test, the fixed effect model was selected. According to the results of the Wald and LR tests, it was considered that the SDM had the best fitting effect, and the estimation results are shown in Table 7. Although the estimation coefficients of each factor were different after replacing the matrix, the significance and direction of the core explanatory variables were consistent with the original results. The properties of each control variable did not change greatly, which verifies the significance of the benchmark model in this paper.

## 5. Conclusions and Implications

Under the new pattern of dual-cycle development with China’s domestic cycle as the core, inter-regional industrial transfers would inevitably show a larger scale and trend, becoming a developmental strategy promoted by the central and local governments. It is of great application value to explore the improvement or damage to residents’ well-being caused by an industrial transfer. Based on the panel data of 30 provinces and cities in China from 2004 to 2019, this study used the spatial panel model to test the specific impact of undertaking an industrial transfer on the improvement of residents’ well-being. On the basis of testing the spatial correlation of residents’ well-being, the article also focused on the moderating effect of environmental regulation on the relationship between undertaking an industrial transfer and the improvement of residents’ well-being. The results show that: 1. At the national level, undertaking an industrial transfer would reduce the well-being of residents. The impact of the inter-regional industrial transfer on the well-being of residents is still in the throes of the stage, and industrial transfers lack effective environmental regulatory constraints.; 2. In terms of regions, industrial transfers improve the well-being of residents in the eastern and central regions effectively. However, in the western region where industries are transferred in, undertaking an industrial transfer had a significant negative effect on the improvement of residents’ well-being. The effect of the industrial transfer-in and industrial transfer-out on residents’ well-being is quite different.; 3. From the perspective of the regulating effect of environmental regulation, environmental regulation reduces the negative impact of the industrial transfer on the well-being of residents significantly. Environmental regulation itself also improves the well-being of residents, which proves the effectiveness of environmental regulations. Moreover, technological progress, government interventions, unemployment, and openness all have significant effects on residents’ well-being.

In view of the above conclusions, this study provides the following policy recommendations for China and other developing countries implementing industrial transfers: 1. Guide local governments to carry out industrial undertakings and transfers orderly according to the local conditions and their own advantages. Create long-term industrial development plans and industrial development positions, and carry out differentiated and coordinated development in various regions.; 2. Provide full play to the public’s supervisory role over the industrial transfer, concentrating on protecting and improving the interests of residents in the process of the industrial transfer. Always pay attention to the people’s employment needs and the need for a better living environment.; 3. Strengthen the central environmental supervision and local territorial management, and select various environmental policy tools reasonably. Stop pollution-intensive industries, eliminate backward production capacities resolutely, and stop underdeveloped areas from becoming a haven for pollution industries in developed areas.; 4. Improve the occupation and employment skills of residents in the industrial transfer-in areas, and enhance the matching degree of talent to undertake an industrial transfer. Promote the agglomeration of a high-quality labor force and the transfer of surplus labor forces to let the undertaking of an industrial transfer and the improvement of residents’ income be integrated deeply.; 5. For most developing countries, short-term, medium-term, and long-term industrial transfer policies should be formulated and implemented carefully. Strict environmental regulations should be adopted for the introduced enterprises, and policy support should be provided to help the transformation and upgrading of enterprises and promote the common development of government, enterprises, and residents.

## Figures and Tables

**Table 1:** Descriptive statistics of the main variables.

Variable	Sample Size	Mean	Standard Deviation	Minimum	Maximum
walf	480	0.6372	0.0593	0.4812	0.8002
indus	480	0.0000	0.9989	−4.4274	4.0863
regul	480	0.5340	0.5291	0.0000	2.5833
unemp	480	0.0349	0.0069	0.0121	0.0630
consum	480	0.4781	0.1398	0.2666	1.2946
innova	480	0.0333	0.0468	0.0000	0.2360
gov	480	0.1457	0.0906	0.0302	0.6209
open	480	0.0242	0.0210	0.0001	0.1210
lumin	480	0.9228	1.9010	0.0051	12.6456
tele	480	0.1159	0.0568	0.0195	0.3198
stru	480	0.4367	0.0835	0.1599	0.6200

**Table 2:** Global Moran’s I statistical indicators for the well-being of residents in 30 provinces of China, 2004–2019.

	2004	2005	2006	2007	2008	2009	2010	2011
Moran’s I	−0.015	0.025	0.009	0.042	0.032	0.053	0.068	0.097
*p*-value	0.102	0.056	0.093	0.020	0.036	0.007	0.003	0.000
	**2012**	**2013**	**2014**	**2015**	**2016**	**2017**	**2018**	**2019**
Moran’s I	0.101	0.107	0.069	0.103	0.111	0.122	0.134	0.130
*p*-value	0.000	0.000	0.002	0.000	0.000	0.000	0.000	0.000

**Table 3:** OLS regression results with different spatial models.

Variable	OLS	SLM	SEM	SDM
indus	−0.0007	−0.0020 *	−0.0025 **	−0.0022 *
unemp	−3.1970 ***	−1.5800 ***	−2.0400 ***	−1.4210 ***
consum	−0.2190 ***	−0.0719 ***	−0.0754 ***	−0.0305
innova	0.0367	0.1810 **	0.2360 ***	0.1570 *
gov	0.1380 ***	0.0297	0.0305	−0.0307
open	−0.8920 ***	−0.2830 ***	−0.3410 ***	−0.2260 **
lumin	0.0023	0.0095 ***	0.0066 ***	0.0084 ***
tele	0.2640 ***	−0.1920 ***	−0.3620 ***	−0.2430 ***
stru	−0.0367	0.0900 ***	0.0601	0.1160 ***
Constant	0.837 ***			
W*indus				0.0101 **
W*unemp				−0.4060
W*consum				−0.0998 *
W*innova				−1.1210 ***
W*gov				0.3860 ***
W*open				−0.2460
W*lumin				0.0062 *
W*tele				0.4060 ***
W*stru				−0.2550 ***
*ρ*		0.4620 ***		0.3140 ***
R^2^	0.7260	0.7130	0.7020	0.7120
*n*	480	480	480	480

**Annotation**: * *p* < 0.1, ** *p* < 0.05, *** *p* < 0.01.

**Table 4:** Analysis of the relationship between an industrial transfer and well-being considering the regional differences.

Variable	Eastern Region	Northeast Region	Central Region	Western Region
indus	−0.0031 **	0.0136 ***	0.0024 *	−0.0013
unemp	−1.6470 **	−1.2120	−2.1600 ***	1.3200 **
consum	−0.0596	0.2740 *	−0.0815	−0.0270
innova	0.0815 *	−1.7010	−0.3840 **	−3.6610
gov	−0.3640	−0.9370 **	0.2420	0.0378
open	0.2060	−0.1430	−0.2790	−0.5180 *
lumin	0.0009	−0.1760 **	−0.0045	−0.0491
tele	−1.3720 ***	−0.3780 **	0.1120	−0.3750 ***
stru	−0.0710	−0.8070 ***	−0.0073	0.0205
W*indus	−0.0030 *	0.0082	0.0010 **	0.0262 *
W*unemp	1.2230	−3.7290	−2.0150 *	7.3500
W*consum	−0.3360 ***	0.1450	−0.0439	0.1200
W*innova	−0.8270 ***	−3.9110 **	0.5540	0.2590
W*gov	1.1110 ***	−0.1200	0.3230	0.7840 **
W*open	0.9080 **	0.1550	0.3750	−0.4000
W*lumin	−0.0038	−0.2080	0.0122	−0.0161
W*tele	1.4520 ***	0.4970 **	−0.1240	−0.6730 *
W*stru	−0.8630 ***	−1.0680 ***	−0.0290	0.1070
*ρ*	0.2090 ***	−0.3580 **	0.3030 ***	−0.1190
*n*	160	48	96	176

**Annotation**: * *p* < 0.1, ** *p* < 0.05, *** *p* < 0.01.

**Table 5:** Direct effect, spatial spillover effect, and the total effect of the SDM model by region.

Variable	Eastern Region			Northeast Region		
	Direct	Indirect	Total	Direct	Indirect	Total
indus	−0.003 **	−0.004	−0.008 **	0.013 ***	0.003	0.016 *
unemp	−1.603 **	1.010	−0.593	−0.532	−3.318	−3.850
consum	−0.080	−0.410 ***	−0.490 ***	0.273 **	0.070	0.342
innova	0.011	−0.980 ***	−0.970 ***	−1.007	−3.100 **	−4.108 *
gov	−0.273	1.246 ***	0.973 ***	−0.970 ***	0.116	−0.858
open	0.291	1.115 **	1.406 **	−0.187	0.243	0.056
lumin	0.001	−0.004	−0.004	−0.147 **	−0.161	−0.308
tele	−1.30 ***	1.416 ***	0.119	0.308 **	0.354 *	0.662 **
stru	−0.140	−1.050 ***	−1.190 ***	−0.650 ***	−0.720 ***	−1.400 ***
	**Central Region**			**Western Region**		
	**Direct**	**Indirect**	**Total**	**Direct**	**Indirect**	**Total**
indus	0.003 *	0.002 *	0.005 *	0.002	−0.025 ***	−0.023 **
unemp	−2.500 ***	−3.500 **	−6.000 ***	1.084 *	6.660 ***	7.744 ***
consum	−0.080 *	−0.097	−0.180 *	−0.028	0.121	0.093
innova	−0.340 *	0.600	0.261	−3.700 ***	0.624	−3.069 **
gov	0.288 *	0.514 *	0.802 **	0.012	0.720 ***	0.733 ***
open	−0.244	0.380	0.136	−0.490 *	−0.370	−0.864
lumin	−0.003	0.015	0.012	−0.049	−0.009	−0.058
tele	0.096	−0.111	−0.015	−0.400 ***	−0.56 **	−0.960 ***
stru	−0.002	−0.045	−0.047	0.022	0.101	0.123

**Annotation**: * *p* < 0.1, ** *p* < 0.05, *** *p* < 0.01.

**Table 6:** Moderating effect test of environmental regulation.

Variable	First	Second	Third
indus	−0.0022 *	−0.0792 ***	−0.0006 *
unemp	−1.4210 ***	4.5330	−3.6990 ***
consum	−0.0305	−1.0940 ***	−0.1350 ***
innova	0.1570 *	4.1680 ***	0.0364
gov	−0.0307	−1.9800 ***	0.1030 ***
open	−0.2260 **	−5.3170 ***	−0.5320 ***
lumin	0.0084 ***	−0.0142	0.0000
tele	−0.2430 ***	1.0840	0.2900 ***
stru	0.1160 ***	1.2350 ***	−0.0852 **
regul			0.0181 ***
W*indus	0.0010	0.0325	0.0037
W*unemp	−0.4060	12.4400	1.0910
W*consum	−0.0998 *	0.4560	0.3260 ***
W*innova	−1.1210 ***	−0.3320	0.0973
W*gov	0.3860 ***	−0.8520	−0.3050 ***
W*open	−0.2460	−9.2590 **	−0.8460 *
W*lumin	0.0062 *	−0.0412	−0.0119 ***
W*tele	0.4060 ***	2.1920	−1.0610 ***
W*stru	−0.2550 ***	2.2750 **	−0.4030 ***
W*regul			0.0841 ***
*ρ*	0.3140 ***	−0.5380 ***	0.2190 ***
R^2^	0.7120	0.6980	0.7370
*n*	480	480	480

**Annotation**: * *p* < 0.1, ** *p* < 0.05, *** *p* < 0.01.

**Table 7:** Robustness test results of the spatial weight matrix based on the geographical location.

Variable	Nationwide	Eastern	Northeast	Central	Western
indus	−0.0040 ***	−0.0030 *	0.0039	0.0036 ***	−0.0055 *
unemp	−2.2250 ***	−1.5390 *	−1.2990 *	−2.7270 ***	−1.4720 **
consum	−0.0997 ***	0.2530 ***	0.2830 **	−0.0652	−0.0447
innova	0.1010 **	0.0261	−1.2350	−1.4360 ***	−0.6460
gov	0.0906 ***	−0.4120 *	−0.3170	0.2300 *	0.1000 **
open	−0.7890 ***	−0.1570	−0.2140 *	0.4870 *	−0.6310 **
lumin	0.0005	0.0029	0.0346	0.0355 **	0.0549 **
tele	0.2050 ***	−0.8440 ***	−0.1490 **	0.7340 ***	−0.0176
stru	0.0434	0.0950	−0.6900 ***	0.0094	0.0663
W*indus	0.0087 ***	0.0056 **	−0.0114 **	0.0030	0.0011
W*unemp	−6.6880 ***	2.0600	−1.8550	−2.6360 ***	−1.2630
W*consum	0.0297	0.6210 ***	0.2930 *	−0.1430 **	−0.1270 ***
W*innova	−0.0121	0.2540**	−2.1520	−0.1040	−0.7380
W*gov	0.0139	−0.2550	0.4190	0.2310	0.1660 **
W*open	0.5040 *	0.4760	0.0705	−0.2970	−1.7520 ***
W*lumin	0.0079 **	0.0150 ***	0.1930 **	−0.0805 ***	−0.0578
W*tele	0.3610 ***	−2.5520 ***	−0.0934	−0.9020 ***	0.3240 **
W*stru	0.1470 **	−0.0658	−0.7120 ***	−0.2350 **	−0.1640 *
*ρ*	0.2150 ***	−0.2730 ***	−0.1910	0.1770 *	0.2200 ***
*n*	480	160	48	96	176

**Annotation**: * *p* < 0.1, ** *p* < 0.05, *** *p* < 0.01.

## Data Availability

We can find these data on the website of www.stats.gov.cn, accessed on 16 November 2022. We also get some data from cgss@ruc.edu.cn.
